# Zebrafish Numb and Numblike Are Involved in Primitive Erythrocyte Differentiation

**DOI:** 10.1371/journal.pone.0014296

**Published:** 2010-12-13

**Authors:** Erica Bresciani, Stefano Confalonieri, Solei Cermenati, Simona Cimbro, Efrem Foglia, Monica Beltrame, Pier Paolo Di Fiore, Franco Cotelli

**Affiliations:** 1 Dipartimento di Biologia, Università degli Studi di Milano, Milano, Italy; 2 The FIRC Institute for Molecular Oncology Foundation (IFOM) at the IFOM-IEO Campus, Milano, Italy; 3 Dipartimento di Scienze Biomolecolari e Biotecnologie, Università degli Studi di Milano, Milano, Italy; 4 European Institute of Oncology (IEO), Milano, Italy; 5 Dipartimento di Medicina, Chirurgia ed Odontoiatria, Università degli Studi di Milano, Milano, Italy; Istituto Dermopatico dell'Immacolata, Italy

## Abstract

**Background:**

Notch signaling is an evolutionarily conserved regulatory circuitry implicated in cell fate determination in various developmental processes including hematopoietic stem cell self-renewal and differentiation of blood lineages. Known endogenous inhibitors of Notch activity are Numb-Nb and Numblike-Nbl, which play partially redundant functions in specifying and maintaining neuronal differentiation. *Nb* and *Nbl* are expressed in most tissues including embryonic and adult hematopoietic tissues in mice and humans, suggesting possible roles for these proteins in hematopoiesis.

**Methodology and Principal Findings:**

We employed zebrafish to investigate the possible functional role of Numb and Numblike during hematopoiesis, as this system allows a detailed analysis even in embryos with severe defects that would be lethal in other organisms. Here we describe that *nb/nbl* knockdown results in severe reduction or absence of embryonic erythrocytes in zebrafish. Interestingly, *nb/nbl* knocked-down embryos present severe downregulation of the erythroid transcription factor *gata1*. This results in erythroblasts which fail to mature and undergo apoptosis. Our results indicate that Notch activity is increased in embryos injected with *nb/nbl* morpholino, and we show that inhibition of Notch activation can partially rescue the hematopoietic phenotype.

**Conclusions and Significance:**

Our results provide the first *in vivo* evidence of an involvement of Numb and Numblike in zebrafish erythroid differentiation during primitive hematopoiesis. Furthermore, we found that, at least in part, the *nb/nbl* morphant phenotype is due to enhanced Notch activation within hematopoietic districts, which in turn results in primitive erythroid differentiation defects.

## Introduction

The formation of blood cells is characterized by a balance between self-renewing multipotent hematopoietic stem cells (HSCs) and differentiated blood elements. All vertebrates display two successive waves of hematopoiesis, known as primitive and definitive hematopoiesis, which take place in anatomically distinct sites [1]. During zebrafish embryonic development, primitive hematopoiesis is mainly limited to erythropoiesis, with some primitive macrophages also being produced. This myeloid population originates from the anterior lateral mesoderm (ALM), while early erythroid precursors originate from two bilateral stripes in the posterior lateral mesoderm (PLM) around the 5-somite stage (ss) [1]. During somitogenesis, these two stripes migrate and converge to the midline and fuse together, forming the intermediate cell mass (ICM), the equivalent of the mammalian yolk sac blood islands, at about 20-ss. Within the ICM, proerythroblasts differentiate and then enter the circulation at 24–26 hours post fertilization (hpf). Subsequently, they mature into primitive erythrocytes, which retain the nucleus and develop a characteristic lentiform shape. At later developmental stages, definitive hematopoiesis produces long-term hematopoietic stem cells able to generate differentiated blood cells of the erythroid, myeloid and lymphoid lineages [1].

The genetic program that drives primitive hematopoiesis is evolutionarily conserved among vertebrates. It has been demonstrated that Notch signaling, which is implicated in cell fate determination in various developmental processes, plays a crucial role in HSCs self-renewal and in the differentiation of blood lineages, both *in vitro* and *in vivo* [2], [3], [4].

One known endogenous inhibitor of Notch activity is the evolutionarily conserved adaptor protein Numb [5], [6]. In mice, several lines of evidence suggest that Numb (Nb) and its homologue Numblike (Nbl) play partially redundant functions in specifying and maintaining neuronal differentiation [7]. The expression of m-*Numb* and m-*Numblike* has been detected in most of the tissues of developing embryos, including the central nervous system (CNS) [8]. In particular, by whole-mount immunostaining m-Numb expression has been detected in the yolk sac of E 7.5–E 8.5 stage mouse embryos, concomitant with primitive erythropoiesis [2]. Moreover, both Numb and Numblike are expressed in adult hematopoietic tissues such as the thymus, spleen and lymph nodes, in both mice and humans [9], [10], [11] and in HSCs isolated from mouse bone marrow [12]. Taken together, these findings therefore raise the possibility that Numb and Numblike proteins might play a role in the embryonic and adult hematopoietic systems.

To address this possibility, Wilson and colleagues investigated the involvement of Numb and/or Numblike in hematopoietic stem cell self-renewal and T cell fate specification in postnatal and adult mice deleted for both *Numb* and *Numblike* in the bone marrow [12]. The absence of both Numb and Numblike did not produce any effect on HSCs self-renewal or T-cell lineage determination, leading the authors to conclude that both Numb and Numblike are dispensable for hematopoiesis in adult mice [12]. However, recent *in vitro* approaches, using hemangioblast-derived blast cell colonies, provided evidence that Numb can modulate the specification of primitive erythrocytes through its interaction with Notch [2].

*Numb* and *Numblike* homologs have been identified and cloned in zebrafish. Numb is ubiquitously expressed during blastula and gastrula stages [13]. Its expression becomes concentrated at the midline at the beginning of somitogenesis and, by the 18-ss, a strong signal is found at the midline from the head to the tail region, and in the retina. At later stages (30-ss), its expression is restricted in the fore-, mid-, and hindbrain and in the eyes [13]. The expression of *numblike* has been detected by whole-mount *in situ* hybridization (WISH) in all regions of zebrafish embryos from 3 hpf until 24 hpf when its expression becomes restricted to the central nervous system [14].

The zebrafish animal model is an ideal organism to study hematopoiesis. Zebrafish embryos are fertilized externally and optically clear, thus, blood cell formation and circulation can easily be assessed throughout development. Moreover zebrafish embryos can develop normally for several days in the absence of blood circulation [15]. Thus, we employed zebrafish to investigate the possible roles of Numb and Numblike during hematopoiesis. Here we report that the simultaneous knockdown of both *numb* and *numblike* produces embryos in which circulating blood cells are absent or severely reduced at 26–28 hpf, when circulation begins. Moreover, the mildest phenotypes we oberserved were characterized by erythroblasts that entered the circulation correctly but were partially impaired in terminal differentiation. Taken together, these results provide the first *in vivo* evidence of the involvement of Numb and Numblike in erythrocyte differentiation during primitive hematopoiesis.

## Results and Discussion

### The knockdown of *numb* and *numblike* results in hematopoietic defects

**Figure 1:**
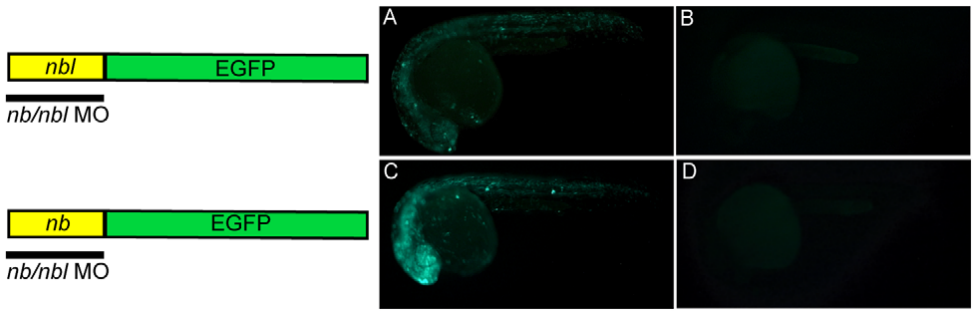
The morpholino can block the translation of both *nb* and *nbl* transcripts. Live zebrafish embryos at 24 hpf. Mosaic EGFP expression in embryos injected at the 1–2 cell stage with 10 pg/nl *nbl*pEGFP reporter construct (A) or 12.5 pg/nl *nb*pEGFP (C). No EGFP signal is detectable in embryos co-injected with 40 pg/embryo *nbl*pEGFP and 0.8 pmol/embryo *nb/nbl* MO (B) or 50 pg/embryo *nb*pEGFP and 0.8 pmol/embryo *nb/nbl* MO (D).

To determine the function of Numb and Numblike during zebrafish development, we performed knockdown experiments using an antisense morpholino [16] which targets the region surrounding the translation start codon of both transcripts (*nb/nbl* MO). In order to confirm that the morpholino could actively knock down the expression of both Numb and Numblike we generated two reporter constructs in which the sequences of *nb* or *nbl* targeted by the MO were fused to the coding sequence of the Enhanced Green Fluorescent Protein (*nb*pEGFP or *nbl*pEGFP). Microinjection of the individual constructs in 1–2 cell stage embryos produced a mosaic EGFP expression (Figure 1 A, C), whereas co-injection of *nb*pEGFP or *nbl*pEGFP with the *nb/nbl* MO resulted in complete loss of the EGFP signal (24 hpf), demonstrating that our *nb/nbl* MO is able to block the production of both Numb and Numblike proteins (Figure 1 B, D).

**Figure 2:**
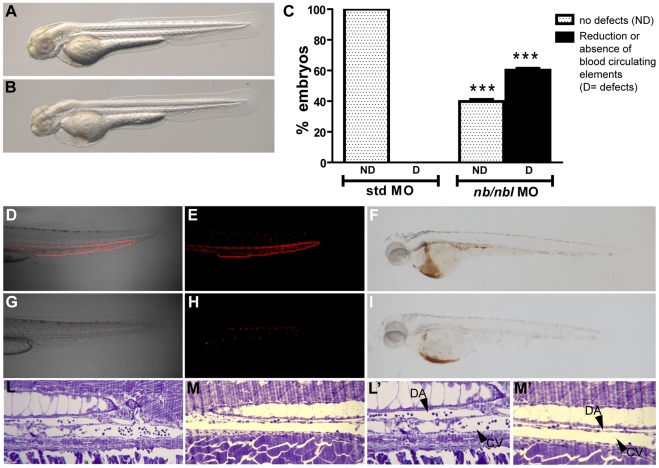
The *nb/nbl* knockdown affects erythrocytes development. A–B. The general morphology of 48 hpf *nb/nbl* morphants is substantially unaffected (B) when compared to control embryos (A). C. Hematopoietic defects in *nb/nbl* morphants at 48 hpf: ∼60% of the *nb/nbl* morphants displayed a severe hematopoietic defect (n = 191). 100% of control embryos was unaffected (n = 93). *** p<0.001 vs std MO. D–I. Detailed images of the blood flow in the trunk-tail region of 48 hpf Tg(*gata1*:dsRed) embryos injected with std MO (0.8 pmol/embryo; D, E) and *nb/nbl* MO (0.8 pmol/embryo; G, H). In *nb/nbl* morphants no circulating red cells are detectable within the trunk vasculature. Whole embryo o-dianisidine staining on 48 hpf std MO embryos (F) and *nb/nbl* morphants (I). L–M′. Longitudinal semithin plastic sections of std MO (L, L′) and *nb/nbl* MO-injected embryos (M, M′) at 2 dpf. Fewer elements are detectable within the Dorsal Aorta (DA) and the Caudal Vein (CV) in *nb/nbl* morphants when compared to control embryos. L′, M′: higher magnification of L, M.

At the selected dose (0.8 pmol/embryo), *nb/nbl* MO-injected embryos (morphants) showed an overall normal morphology with no visible alteration in the patterning of the CNS. However, no blood elements entered the circulation at 26–30 hpf, as occurred in control injected embryos (data not shown). At 48 hpf, the morphology of *nb/nbl* MO-injected embryos appeared preserved (Figure 2 A, B) but the majority of the *nb/nbl* morphants displayed reduced motility, likely referable to nervous system defects (data not shown). At this stage of development, blood cells were actively circulating in control embryos (Figure 2 D, E; Figure S1 A, C; e.g., Video S1) but 27% of the *nb/nbl* morphants (52/191 embryos) showed no circulating blood cells (Figure 2 G, H; Figure S1 B, D; e.g., Video S2), and an additional 33% (63/191 embryos) displayed only a few circulating blood cells (e.g., Video S3). Thus, around 60% of the *nb/nbl* morphants displayed a severe hematopoietic defect (Figure 2 C; Figure S1 A–D). At 3 days post fertilization (dpf), affected embryos developed pericardial edema, which became more pronounced all over the yolk sac at 4–6 dpf; the most severely affected *nb/nbl* morphants died by this stage of development. Nevertheless *nb/nbl* morphants treated starting from 3 dpf with mannitol (250 mM), which prevents systemic edema by eliminating the osmotic water gradient [17], displayed only limited cardiac edema (data not shown) suggesting that these defects might be at least in part due to a secondary effect caused by the reduction/absence of blood circulation. Since Notch is known to be involved in cardiac development [18], [19], we could not exclude the presence of cardiac defects in our *nb/nbl* morphants. However, by microangiography we showed that at 2 dpf even in *nb/nbl* morphants injected at high dose (1 pmol/embryo) the heart functionality is not drastically compromised and the axial vasculature is patent (Figure S2). Furthermore, we analyzed vessel morphology in 2 dpf *nb/nbl* MO-injected embryos by longitudinal semi-thin plastic sections. In *nb/nbl* morphants the morphology of the axial vasculature appeared preserved, although only few blood elements were detectable within the lumen of the vessels (Figure 2 L, L′, M, M′). This confirms our *in vivo* analysis which showed a morphologically intact vasculature.

Overall, these results strongly argue that Numb and Numblike play a role in zebrafish primitive hematopoietic development.

### Functional knockdown of *numb* and *numblike* cause primitive erythrocyte hypoplasia

To assess the presence of differentiated primitive erythrocytes in *nb*/*nbl* morphants we analyzed the hemoglobin content by whole embryo o-dianisidine staining. The presence of the erythrocytes in 48 hpf *nb*/*nbl* injected embryos (0.8 pmol/embryo) appeared strongly reduced (67%; Figure S1 H) and only few differentiated red blood cells were detectable within the *sinus venosus* (Figure 2 F, I). Higher doses of *nb*/*nbl* MO (0.9–1 pmol/embryo) increased the hematopoietic phenotype in a dose-dependent manner (Figure S1 H). Complete loss of hemoglobin staining was observed in embryos injected with higher doses of *nb*/*nbl* MO (0.9 pmol/embryo and 1 pmol/embryo; Figure S1 E–H). However, the morphology of *nb*/*nbl* morphants resulted less preserved at these doses. Therefore we decided to perform the molecular characterization of the hematopoietic phenotype in embryos injected with a lower dose of *nb*/*nbl* MO (0.8 pmol/embryo).

**Figure 3:**
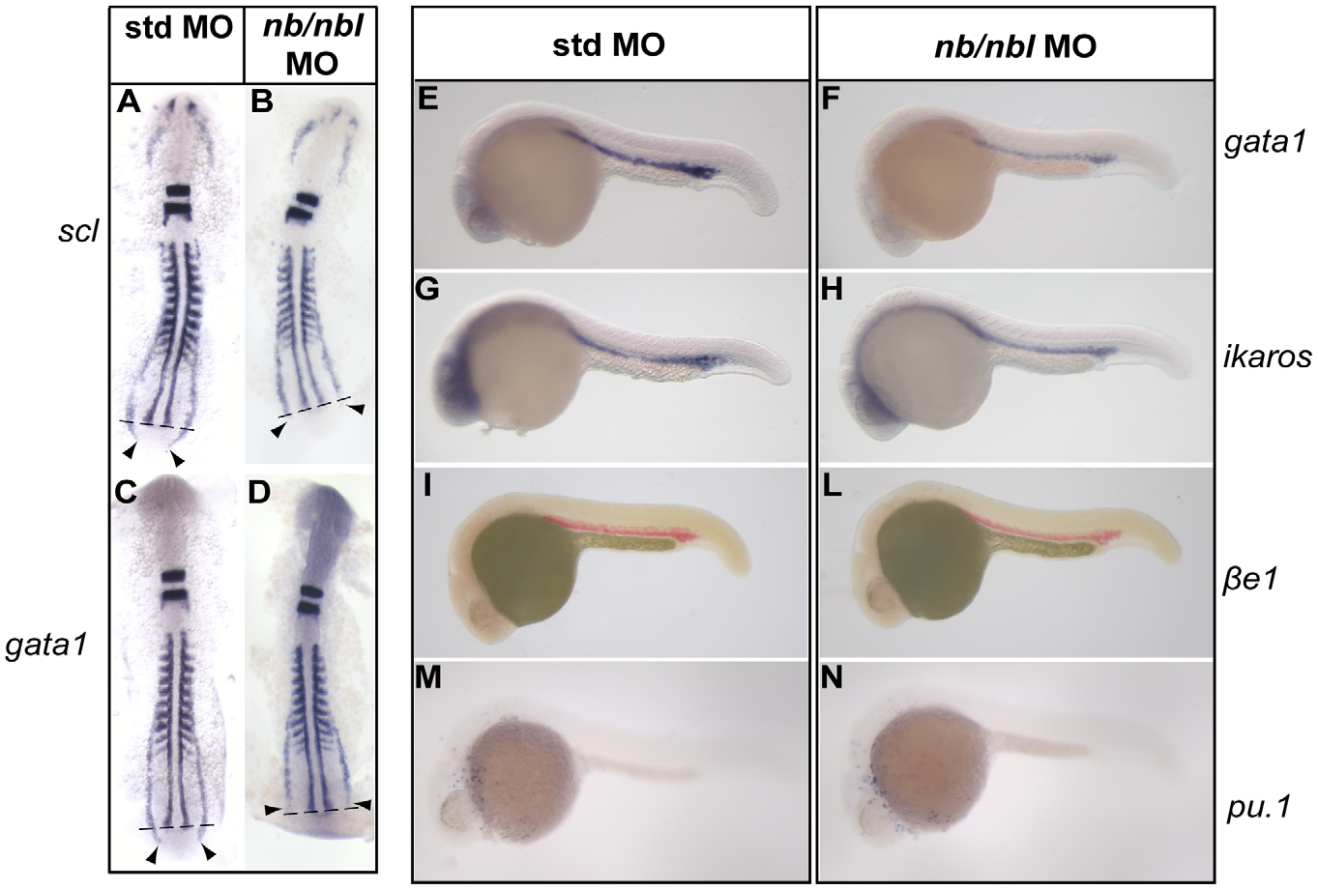
Expression of hematopoietic genes in *nb/nbl* morphants. A–D. Flat mounted 10-ss embryos. Triple WISH were performed on std MO (A, C) and *nb/nbl* MO-injected embryos (B, D). The expression of *myoD* in somitic and pre-somitic mesoderm appeared unaltered in *nb/nbl* MO embryos (B, D). The expression of *krox20* in the rombomeres 3 and 5 is unaffected in *nb/nbl* morphants (B, D). A–B. The expression of *scl* appears reduced in the posterior PLM (black arrowheads). C–D. *gata1* expression is reduced in *nb/nbl* morphants, particularly in the posterior PLM (black arrowheads). E–N. Lateral view of 22–24 hpf std MO embryos (E, G, I, M) and *nb/nbl* morphants (F, H, L, N). In *nb/nbl* morphants at 22 hpf *gata1* expression is reduced in the ICM (F); *ikaros* and *βe1* globin are also downregulated at 24 hpf (H, L). The myeloid marker *pu.1* is expressed at normal levels in *nb/nbl* MO-injected embryos (N).

Next, we analyzed the expression pattern of several hematopoietic markers by WISH, to gain insight on the molecular mechanisms responsible for derailing primitive hematopoiesis in *nb/nbl* morphants. The expression of the transcription factor *fli1*, which marks both blood and endothelial precursors within the ALM and PLM, appeared substantially unaffected in *nb/nbl* morphants at 8–10-ss (data not shown). However, at 10-ss, the stem cell leukemia gene *scl*, which is also expressed in hematopoietic and vascular progenitors, was reduced in the posterior region of the PLM (Figure 3 A, B).

The erythroid transcription factor *gata1*, initially expressed at early somitogenesis stages in a subset of *scl*+ cells in two bilateral stripes of the PLM [20], plays a key role in erythroid lineage commitment and is considered the first marker of erythroid progenitor cells. The *gata1*+ cells, which migrate to the midline during somitogenesis stages, develop into proerythroblasts within the ICM at about 20-ss [20]. *nb/nbl* knocked-down embryos showed a reduction of *gata1* expression domain particularly in the posterior region of the PLM at 10-ss (Figure 3 C, D); moreover, at 22 hpf, *gata1* expression was reduced at the level of the ICM (Figure 3 E, F). Similarly to *gata1*, the HSC marker *ikaros* and the erythroid specific *βe1 globin* gene within proerythroblasts were downregulated in the ICM around 24 hpf (Figure 3 G–L). Notably, injection of an higher dose of *nb*/*nbl* MO (1 pmol/embryo) shows a dose dependent reduction of both *gata1* and *βe1 globin* expression within the ICM at 22–24 hpf (Figure S3).

It is well established that the interplay between *gata1* and *pu.1*, which drives the development of myeloid cells, is essential to establish the myelo-erythroid progenitor cell fate during zebrafish primitive hematopoiesis. It has been reported that loss of *gata1* results in *pu.1* ectopic expression within the ICM at 22–24 hpf converting erythropoiesis into myelopoiesis [21]. We therefore assessed the onset of myeloid lineage on 24 hpf *nb/nbl* morphants. The expression of *pu.1* across the yolk sac of the *nb/nbl* morphants was unaltered and no *pu.1*+ cells were detectable in the ICM (Figure 3 M, N). Based on these observations, we concluded that, in *nb/nbl* MO-injected embryos, myeloid lineage specification occurs normally and that, in our *nb/nbl* morphants, downregulation of *gata1* does not result in primitive erythroid precursor conversion into myeloid cells.

**Figure 4:**
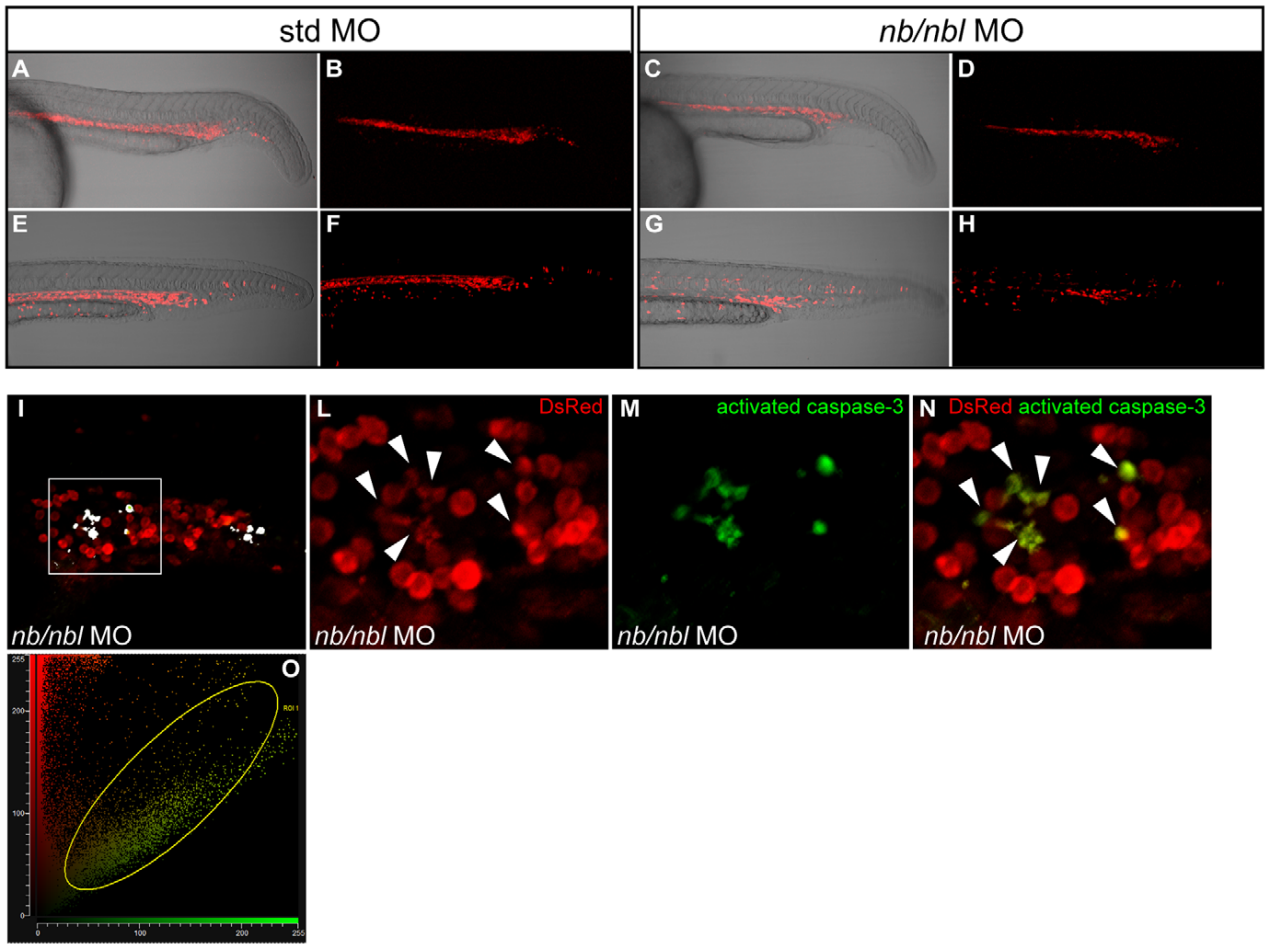
dsRed+ erythroid cells are dramatically reduced at 28–30 hpf. A–H. Tg(*gata1*:dsRed) embryos injected with std MO (A, B, E, F) and *nb/nbl* MO (C, D, G, H) were examined by confocal microscopy between 24–30 hpf. Fluorescent images (B, D, F, H) were merged with bright field images (A, C, E, G). In *nb/nbl* morphants at 24–26 hpf red fluorescent erythroid cells are present within the ICM (C, D) but at 28–30 hpf the overall fluorescence of *nb/nbl* morphants appears strongly reduced (G, H). I–N. Whole-mount double immunofluorescence on Tg(*gata1*:dsRed) *nb/nbl* morphants at 28–30 hpf to detect caspase-3 activation (green) and DsRed (Red). Single optical section of *nb/nbl* morphants obtained by confocal microscopy (20× magnification, I). I. White spots, indicating double positive cells, have been pseudocoloured according to the region of interest (ROI1 in O). The sub-image area, shown in detail in panels L–N, is highlighted by the white box. L–N. Insets of single channel fluorescent images (L, M) and merge (N) are shown. Activated caspase-3 overlaps with some dsRed+ cells (white arrowheads, L). O. Fluorogram shows the degree of colocalization between red signals (dsRed) and green signals (activated caspase-3); colocalization is indicated by the region of interest (ROI1).

Since zebrafish represents an ideal tool for direct *in vivo* observations we decided to gain insight into the erythropoietic defects by monitoring the behavior of *gata+* cells during *nb/nbl* morphant development. Thus, we injected the *nb/nbl* MO into the Tg(*gata1*:dsRed) transgenic line, where the expression of the dsRed fluorescent protein is driven by the *gata1* promoter. At 24–26 hpf, although the overall fluorescence appeared slightly reduced, red fluorescent erythroid cells were present within the ICM in transgenic *nb/nbl* morphants (Figure 4 A–D). However, between 28–30 hpf, when circulation occurs in control embryos, the overall fluorescence of *nb/nbl* morphants appeared to be strongly reduced (Figure 4 E–H). These data suggest that erythroblasts undergo apoptosis after 26 hpf in *nb/nbl* injected embryos.

Taken together these observations suggest that at least part of the erythroid program is initiated in *nb/nbl* MO-injected embryos, in spite of *gata1* downregulation. However, early erythroid cells fail to undergo terminal differentiation. Such a scenario agrees with previous work showing that erythroid cells defective in *gata1* develop normally into proerythroblast but fail to mature properly and undergo apoptosis [22]. To gain further support for this contention, we performed whole-mount immunofluorescence to detect caspase-3 activation at 1-h intervals from 22 hpf to 28–30 hpf in Tg(*gata1*:dsRed) embryos injected with standard MO and *nb/nbl* MO. *nb/nbl* morphants from 22 to 26 hpf revealed no significant increase in cell death within the ICM, when compared to control embryos (data not shown). From 26 to 30 hpf circulation began in control embryos. Conversely at the same stage of development, high levels of dsRed+ cells were still present in the ICM region in *nb/nbl* MO-injected embryos and apoptotic cells became detectable (Figure S4) suggesting that asynchronous apoptotic events occur in these morphants. We then confirmed by confocal microscopy that eryhtroid cells of *nb/nbl* morphants undergo apoptosis around 26–30 hpf in Tg(*gata1*:dsRed) embryos by looking for colocalization of the dsRed+ cells and activated caspase-3 (Figure 4 I–O). The confocal analysis revealed the presence of DsRed+ erythroblasts positive for the activated caspase-3 exclusively in the ICM of *nb/nbl* morphants and not in control embryos (Figure 4 I–O). These findings clearly indicate that primitive erythroid cells of *nb/nbl* morphants fail to differentiate and, instead, undergo apoptosis.

**Figure 5:**
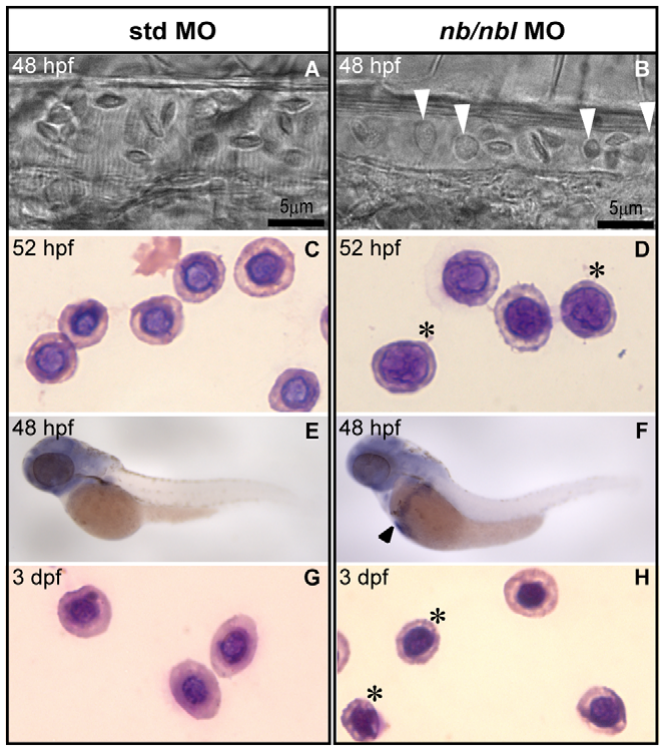
Erythroblasts fail to differentiate into erythrocytes in apparently unaffected *nb/nbl* morphants. A–B. Bright-field microscopy of blood cells in the caudal arteries of living 48 hpf std MO embryos (A) and *nb/nbl* morphants (B). *nb/nbl* MO-injected embryos with no apparent hematopoietic defects show the presence of abnormal-shaped blood cells (white arrowheads) in the blood flow (B). C–D, G–H. Wright-Giemsa staining of circulating embryonic red blood cells from controls and *nb/nbl* morphants at 52 hpf (C, D) and 3 dpf (G, H). Erythroid cells of apparently unaffected *nb/nbl* MO injected embryos were larger, showed a large nucleus and had more basophilic cytoplasm indicating the presence of maturation defects (D, H). Asterisks indicate representative erythroid cells with maturation defects. E–F. WISH using *gata1* on 48 hpf std MO (E) and *nb/nbl* MO-injected embryos (F). *gata1* expression persists in red blood cells of *nb/nbl* morphants revealing the presence of immature red cells (black arrowhead).

Since our findings revealed that primitive erythroid differentiation is affected in *nb*/*nbl* morphants we decided to extend our analysis by investigating erythrocyte maturation directly through screening blood cell morphology *in vivo*. Primitive circulating erythroblasts normally present a rounded shape; between 1.5–2 dpf, they develop into erythrocytes with a lentiform appearance. The *in vivo* analysis of erythrocyte morphology in apparently unaffected *nb*/*nbl* MO-injected embryos at 48 hpf revealed the presence of some round-shaped blood cells (Figure 5 A, B). Wright-Giemsa staining of circulating embryonic red blood cells obtained from control embryos and *nb/nbl* morphants at 52 hpf (Figure 5 C, D) demonstrated that erythroid cells of apparently unaffected *nb/nbl* MO-injected embryos showed generalized maturation defects. To confirm these observations we performed WISH using *gata1* as an early marker of the erythroid lineage. The expression of this gene is normally restricted to erythroid cells and is downregulated at 48 hpf, but in *nb*/*nbl* knocked down embryos, *gata1* expression persisted in red blood cells at 48 hpf (Figure 5 E, F). In order to exclude that the maturation defects of erythroid cells in apparently unaffected *nb*/*nbl* morphants could be due to a generalized developmental delay we performed Wright-Giemsa staining on blood smears at 3 dpf. This analysis showed that also at this developmental stage the maturation defects of the erythroid cells in *nb*/*nbl* MO-injected embryos are still detectable (Figure 5 G, H).

Taken together these data strongly suggest that *numb* and *numblike* are required for normal erythroid differentiation in the zebrafish embryo during primitive erythropoiesis.

### Phenotype specificity

We so far used a morpholino (*nb*/*nbl* MO) able to block the translation of both *numb* and *numblike*. In order to investigate the individual contribution of *numb* and *numblike* to primitive erythropoiesis, we designed two splice-blocking morpholinos (*nb* MO1 , *nbl* MO1; Figure S5) that were injected separately in Tg(*gata1*:dsRed) embryos. We found that injection of either *nb* MO1 (1.4 pmol/embyo) or *nbl* MO1 (0.3 pmol/embryo) into the transgenic line Tg(*gata1*:dsRed) could reproduce the hematopoietic defects observed with *nb/nbl* MO, albeit with lower penetrance (*nb* MO1 ∼19%; *nbl* MO1 ∼25%; Figure S5).

**Figure 6:**
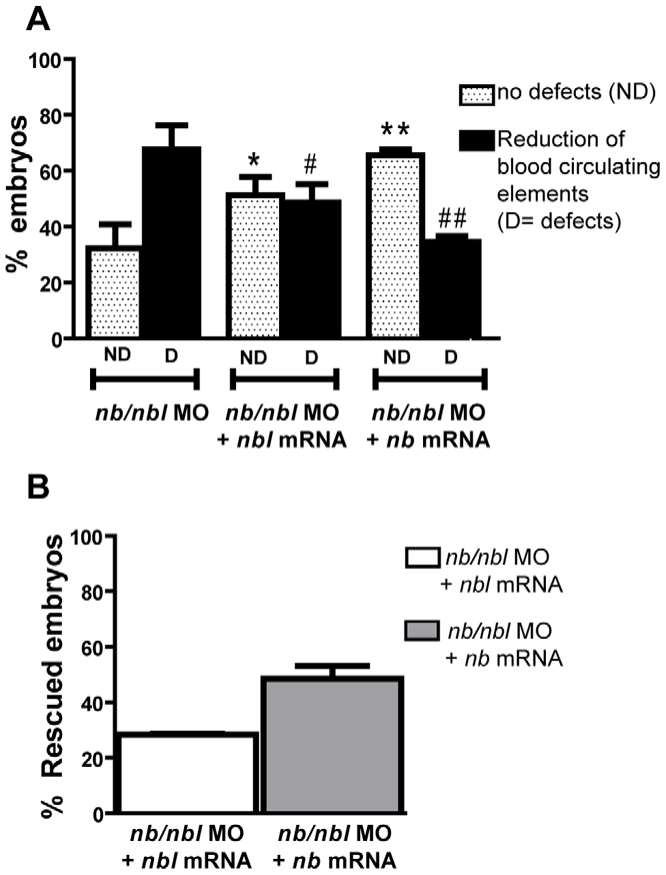
*nb* and *nbl* mRNAs rescue the hematopoietic phenotypes of *nb/nbl* morphants. A–B. Percentages of Tg(*gata1*:dsRed) with hematopoietic defects obtained in rescue experiments (A) and corresponding percentages of embryos which rescue the hematopoietic phenotype (B). Injection of *nb/nbl* MO (0.8 pmol/embryo) produced ∼68% of Tg(*gata1*:dsRed) embryos with hematopoietic defects at 48 hpf (n = 142). Co-injection of 1 ng/embryo of *numb*-EGFP mRNA and 0.8 pmol/embryo *nb/nbl* MO (n = 114) produced ∼35% of embryos with hematopoietic defects (A) that correspond to ∼49% of rescue (B). ∼49% of embryos co-injected with 170 pg/embryo of *numblike* mRNA and 0.8 pmol/e *nb/nbl* MO (n = 105) showed hematopoietic defects (A) corresponding to ∼28% of rescue (B). * p<0.05 vs *nb/nbl* MO no defects, **p<0.01 vs *nb/nbl* MO no defects, #p<0.05 vs *nb/nbl* MO defects, ##p<0.01 vs *nb/nbl* MO defects.

In order to demonstrate that the erythroid defects observed in the *nb/nbl* morphants were specifically due to the *nb/nbl* MO-induced reduction of both Numb and Numblike, we performed rescue experiments in Tg(*gata1*:dsRed) embryos. Co-injection of 1 ng/embryo of *numb*-EGFP mRNA and 0.8 pmol/embryo *nb/nbl* MO resulted in a high rate lethality and produced embryos with severe morphological abnormalities. However at 48 hpf only ∼35% of co-injected embryos showed hematopoietic defects (Figure 6 A); given that ∼68% of *nb/nbl* morphants show hematopoietic defects this correspond to ∼49% of rescue (n = 114; Figure 6 B). On the other hand, the ∼49% embryos co-injected with 170 pg/embryo of *numblike* mRNA and 0.8 pmol/e *nb/nbl* MO showed a reduction of DsRed+ cells (Figure 6 A) indicating that the ∼28% of the embryos rescued the hematopoietic defects (n = 105; Figure 6 B). Based on these results, we concluded that both *numb* and *numblike* control primitive erythropoiesis, with partially overlapping functions.

### Numb ablation and Notch activation in primitive erythropoiesis

To investigate if the loss of function of *numb* and *numblike* influenced the correct activation of the Notch pathway, we tested the expression of a downstream target of Notch. We decided to analyze the homolog of the mammalian gene *HES1*, the Notch target gene *her6*, by WISH because in 8–10-ss control embryos it is expressed not only in the CNS and in the pre-somitic mesoderm but also in the PLM. At the same stage, *nb/nbl* MO-injected embryos displayed an enlarged expression domain of *her6* in particular at the level of the PLM (Figure S6 A, B). These data suggest that the knockdown of both *numb* and *numblike* could result in enhanced Notch activity in hematopoietic districts. Interestingly, it has been reported that HES1 interacts with GATA1 both *in vivo* and *in vitro* and can inhibit erythroid/megakaryocytic differentiation by suppressing GATA1 activity [23]. Moreover, it has also been demonstrated that *gata1* expression undergoes a positive autoregulatory mechanism in zebrafish [24]. Given the functional conservation between homologous proteins and regulatory mechanisms among vertebrates, we can speculate that an alteration in this regulatory pathway could be responsible for the hematopoietic phenotype produced by *nb/nbl* knockdown.

We therefore tried to correct Notch activity levels by treating the *nb/nbl* morphants with a γ-secretase inhibitor (DAPT). γ-secretase executes the final cleavage step required for the activation of Notch. Thus, in principle, its inhibition could revert a possible increase in Notch activity caused by the loss of endogenous Notch inhibition by Numb/Numblike.

It has been reported that wild-type embryos treated from sphere stage (4.3 hpf) with DAPT display somitic and neuronal defects that are typical of Notch-depleted embryos [25]. We tested the effectiveness of the drug by treating wild-type embryos from sphere stage to 24 hpf with 100 µM DAPT and, as expected, we phenocopied the *mind bomb* (*mib*) mutant, which lacks a ubiquitin ligase that is essential for Notch signaling [26]. To avoid gross morphological defects, we tested different concentrations of DAPT (75 µM, 150 µM, 166 µM) on controls and *nb/nbl* morphants treated from 2-ss to 26 hpf, when primitive hematopoietic commitment and determination take place. Control Tg(*gata1*:dsRed) embryos treated with either DMSO or DAPT resulted in mild phenotypic abnormalities reminiscent of *mib* phenotypes (e.g. body curvature, mild head abnormality, sporadic premature venous return) but not in a decrease in dsRed+ cells (data not shown). Our working concentrations were at/or over the limit of solubility for DAPT, making it liable to precipitate out of solution and raising the possibility of nonspecific toxic effects; therefore we could not test higher DAPT concentrations. In all cases the general morphology of *nb/nbl* morphants resulted comparable to control embryos, but a reproducible dose-dependent rescue of the hematopoietic phenotype was observed (respectively: DAPT 75 µM 13% n = 40, DAPT 150 µM 16% n = 106, DAPT 166 µM 18% n = 50; Figure S6 C) making these results worth considering. Our results suggest that, at least in part, the *nb/nbl* morphant phenotype is due to enhanced Notch activation within hematopoietic districts, which in turn results in primitive erythroid differentiation defects.

## Materials and Methods

### Zebrafish lines and maintenance

Current italian national rules: no approval needs to be given for research on zebrafish embryos. Zebrafish were raised and maintained according to established techniques (Westerfield M., 2000. The Zebrafish Book. A guide for the laboratory use of zebrafish (Danio rerio). Eugene: University of Oregon Press, [27]), approved by the veterinarian (OVSAC) and the animal use committee (IACUC) at the University of Oregon, in agreement with local and national sanitary regulations. The following strains were used: wild type AB and TL lines (obtained from the Wilson lab, University College London, London, United Kingdom) and the transgenic line Tg(*gata1*:dsRed) kindly provided by M. Santoro (Molecular Biotechnology Center, University of Torino) was used for general analysis of red cells development.

### Plasmid Construction

To construct *nb*pEGFP and *nbl*pEGFP we cloned the cDNAs encoding the region targeted by *nb/nbl* MO of *numb* and *numblike* into the *NheI–AgeI* sites of pEGFP-C1 vector. *numblike* cDNA fragments were obtained using the following complementary oligos:

5′-CTAGGTGGAGATGAATAAGCTGCGTCAGAGCCTG-3′

5′-CCGGCAGGCTCTGACGCAGCTTATTCATCTCCAC-3′.

*numb* cDNA fragments were obtained using the following complementary oligos:

5′-CTAGTCAGCGATGAATAAGCTACGGCAGAGTTTC-3′

5′-CCGGGAAACTCTGCCGTAGCTTATTCATCGCTGA-3′.

### Morpholinos and synthetic RNAs

Antisense morpholino against both zebrafish *numb* and *numblike* (MO; Gene Tools, Philomath, OR) AUG translation start site region:

*nb/nbl* MO, 5′- CAGGCTCTGACGCAGCTTATTCATC-3′.

Splice MOs against *numb* and *numblike* pre-mRNA were designed as follows:

*nbl-* MO1, 5′-CCCACTCTGAGGGTAGAAAAATTGA-3′;

*nb-* MO1, 5′-CACACAGCAAAACTTACTTTTTTAA-3′.

MOs, diluted in Danieau buffer [16] were injected at the 1- to 2-cell stage. Escalating doses of each MO were tested for phenotypic effects; as control for nonspecific effects, each experiment was performed in parallel with a standard control oligo (std MO) with no targets in zebrafish embryos. For knockdown experiments, we usually injected: 0.8 pmol/embryo, 0.9 pmol/embryo, 1 pmol/embryo of *nb/nbl* MO, 0.3 pmol/embryo *nbl-*MO1, 1.4 pmol/embryo *nb-*MO1.

Sense mRNA encoding full-length *numb* fused to EGFP was transcribed *in vitro* from pCS2+-*numb:EGFP* kindly provided by A. Reugels [13] using mMESSAGE mMACHINE kit (Ambion). Sense mRNA encoding full length *numblike* was transcribed *in vitro* from pCS2+-*numblike* using mMESSAGE mMACHINE kit (Ambion).

### Statistical analysis

Statistical analysis were performed with student's test or one-way ANOVA followed by Dunnetts' post-test when needed using GraphPad PRISM version 5.0 (GraphPad, San Diego, California). A p<0.05 indicates a statistically significant effect.

### Semithin sections

Semithin plastic sections (0.8 µm) on 2 dpf *nb/nbl* MO and std MO-injected embryos were obtained as described [28]. Images were taken using a Leica DM6000 B microscope equipped with a Leica DCF480 digital camera and the LAS Leica imaging software (Leica, Wetzlar, Germany).

### Whole-mount in situ hybridization, immunofluorescence, microangiography and imaging

WISH were carried out essentially as described by Thisse [29].

The following probes were synthesized as described in the corresponding papers: *scl* [30], *gata1* [20], *myod* [31], *krox20* [32], *ikaros* [33], *βe1 globin* [34], *pu.1* [35], *her6* [36]. Images were taken with a Leica MZFLIII epifluorescence stereomicroscope equipped with a DFC 480 R2 digital camera and LAS Leica imaging software (Leica, Wetzlar, Germany). Active caspase-3 detection was performed essentially as described by Kratz [37]. The following antibodies were used: anti-cleaved caspase-3 (Cell signaling #9661) diluited 1∶250 and subsequently incubated with 1∶200 Alexa Fluor 488 goat anti-rabbit IgG (Invitrogen #A31627); anti-RFP (MBL International Corporation #M155-3) diluited 1∶100 and subsequently incubated with 1∶200 Alexa Fluor 555 goat anti-mouse (Invitrogen #A31621). Images were taken with a Leica DM6000 B microscope equipped with a DFC 360 FX digital camera. Confocal microscopy was performed on a Leica TCS SP2 AOBS microscope, equipped with an argon laser. Images were processed using the Adobe Photoshop software (Adobe, San Jose, CA). Movies were processed using the QuickTime Player software (Apple, Cupertino, CA). Microangiography exeriments were performed essentially as previously described [38]. Embryos were injected with Dextran-TMR (tetramethylrhodamine; molecular weight 2×10^6^ Da, Molecular Probes).

### O-dianisidine staining, microscopic observation of circulating blood cells and blood smears

Zebrafish embryos were stained for 15 min in the dark in o-dianisidine staining solution, as previously described [20]. Stained embryos were cleared with benzyl benzoate/benzyl alcohol (2∶1, vol/vol) and were analyzed at Leica MZFLIII epifluorescence stereomicroscope. Bright-field microscopy of blood cells in the caudal arteries of living std MO and *nb/nbl* MO-injected embryos (2dpf) was performed with a Leica DM6000 B microscope equipped with a DFC 360 FX digital camera. Embryonic zebrafish erythrocytes were collected by tail amputation of 8–10 std MO and *nb/nbl* MO-injected embryos at 52 hpf and 3 dpf. Blood smears were performed as previously described [39] and stained with Wright-Giemsa stain (Sigma #WG16).

### Zebrafish DAPT treatments

A 40 mM stock solution of DAPT (γ-secretase inhibitor IX; Calbiochem) in DMSO was diluited in E3 embryo medium to the following concentrations: 75 µM, 150 µM , 166 µM. *nb/nbl* MO and std MO-injected embryos were dechorionated by pronase treatment and treated with DAPT from 1–2-ss to 24–26 hpf at 28°C. As control *nb/nbl* MO and std MO-injected embryos were treated with E3 embryo medium containing the same concentration of DMSO carrier only. The percentage of rescue were calculated taking in account the fraction of Tg(*gata1*:dsRed) *nb/nbl* morphants with hematopoietic defects in DAPT treated *nb/nbl* morphants versus *nb/nbl* morphants with hematopoietic defects in DMSO (controls).

## Supporting Information

Figure S1 Dose dependent hematopoietic phenotype induced by nb/nbl MO. A–D. Tg(gata1:dsRed) std MO and nb/nbl MO (0.8pmol/embryo) at 48 hpf. Images were taken in bright field (A, B) and using a rhodamine emission filter (C, D). E–G. Analysis of the hemoglobin content by whole embryo o-dianisidine staining. 48 hpf std MO embryos (E) and nb/nbl morphants (1 pmol/embryo; F, G). At this dose the 62% of nb/nbl morphants shows a drastic reduction of the hemoglobin content (F), an additional 30% shows complete loss of hemoglobin staining (G). H. Injection of different doses of nb/nbl MO (0.8–1 pmol/embryo) produces a dose-dependent hematopoietic phenotype. The data are referred to a single typical experiment.
(2.29 MB TIF)

Figure S2 The heart functionality and the axial vasculature are not drastically compromised in nb/nbl morphants. Microangiography experiments, were performed on 2 dpf controls (A, B) and embryos injected with nb/nbl MO at the high dose of 1 pmol/embryo (C–F). In nb/nbl morphants (D, F) the injected dye flows into the main axial vessels as in control embryos (B).
(1.06 MB TIF)

Figure S3 Dose dependent reduction of gata1 and βe1 globin expression in nb/nbl morphants. WISH were performed on controls (std MO; A, D) and embryos injected with different doses of nb/nbl MO (0.8 pmol/embryo, B, E; 1 pmol/embryos, C, F). In the ICM of 22–24 hpf nb/nbl MO-injected embryos the downregulation of gata1 and βe1 appears dose dependent.
(0.86 MB TIF)

Figure S4 Caspase-3 activation in nb/nbl morphants at 28–30 hpf. A–B. Whole-mount immunofluorescence to detect caspase-3 activation (green signal), detailed view of the ICM region of 26–28 hpf Tg(gata1:dsRed) embryos injected with std MO (A) and nb/nbl MO (B). C–F. Single optical sections of 26–28 hpf control and nb/nbl MO-injected embryos in which whole-mount immunofluorescence for caspase-3 activation (green signal) was performed. Fluorescent images (C, D) were merged with bright field images (E, F). Detailed view of the ICM region. Caspase-3 activation can be detected in erythroid cells of nb/nbl morphants (white arrowheads; D, F).
(1.15 MB TIF)

Figure S5 Single numb and numblike knockdown reproduce the nb/nbl morphants hematopoietic defects with low penetrance. A–B. Injection of nb MO1 and nbl MO1 specifically blocks splicing of the targeted pre-mRNAs. PCR reactions were performed on cDNAs retrotranscribed from total RNA extracted from 29 hpf nb MO1 injected embryos (1.4 pmol/embryo; A), nbl MO1 injected embryos (0.3 pmol/embryo; B), std MO injected embryos (0.3 pmol/embryo or 1.4 pmol/embryo; A, B). β-actin has been tested as an internal control (data not shown). A control PCR reaction performed without cDNA is shown in lane 3 of both the boxes (A, B). Primers: nb MO1-5′: CACCAGTGGCAGACCGATGAA nb MO1-3′: ACCGCTCGCACAGCCTTCTTA nbl MO1-5′: TCGGGCTGGTGGAGGTGGAT nbl MO1-3′: CCGTCACGGCAGATGTAAGAG. C. Single injection of nb MO1 (1.4 pmol/embryo) or nbl MO1 (0.3 pmol/embryo) in Tg(gata1:dsRed) produces the hematopoietic phenotype respectively in ∼19% (n = 99) and ∼25% (n = 125) of the MO injected embryos (*p<0.05 vs std MO no defects, **p<0.01 vs std MO no defects, #p<0.05 vs std MO defects, ##p<0.01 vs std MO defects). 100% of control embryos was unaffected (n = 65).
(0.95 MB TIF)

Figure S6 her6 is ectopically expressed in nb/nbl morphants. A–B. Posterior view of 8–10-ss embryos. The nb/nbl morphants display an enlarged expression domain of the Notch target gene her6 within the PLM region (B; black arrowheads), when compared to controls (A). C. Percentages of rescue of the hematopoietic phenotype in nb/nbl morphants treated with different concentrations of DAPT.
(1.00 MB TIF)

Video S1 Blood flow in the trunk-tail region of a 48 hpf control embryo. Blood cells were actively circulating in 48 hpf control embryos (std MO 0.8 pmol/embryo). High-magnification bright-field videomicrographs of the mid-trunk of a 48 hpf control embryo were taken at a Leica DM6000 B microscope equipped with a DFC 360 FX digital camera.
(1.29 MB MOV)

Video S2 A 48 hpf nb/nbl morphant with no circulating blood cells. nb/nbl MO-injected embryos (0.8 pmol/embryo; 39/137 embryos) showed no circulating blood cells. High-magnification bright-field videomicrographs of the mid-trunk of a 48 hpf nb/nbl morphant were taken at a Leica DM6000 B microscope equipped with a DFC 360 FX digital camera.
(0.44 MB MOV)

Video S3 A 48 hpf nb/nbl morphant with few circulating blood cells. nb/nbl MO-injected embryos (0.8 pmol/embryo; 44/137 embryos) displayed only a few circulating blood cells. High-magnification bright-field videomicrographs of the mid-trunk of a 48 hpf nb/nbl morphant were taken at a Leica DM6000 B microscope equipped with a DFC 360 FX digital camera.
(0.65 MB MOV)
