# Supplementary figures and images for: Zebrafish Numb and Numblike Are Involved in Primitive Erythrocyte Differentiation
Source: PLoS One. 2010 Dec 13;5(12):e14296. doi: 10.1371/journal.pone.0014296 (PMC3001437; doi:10.1371/journal.pone.0014296)

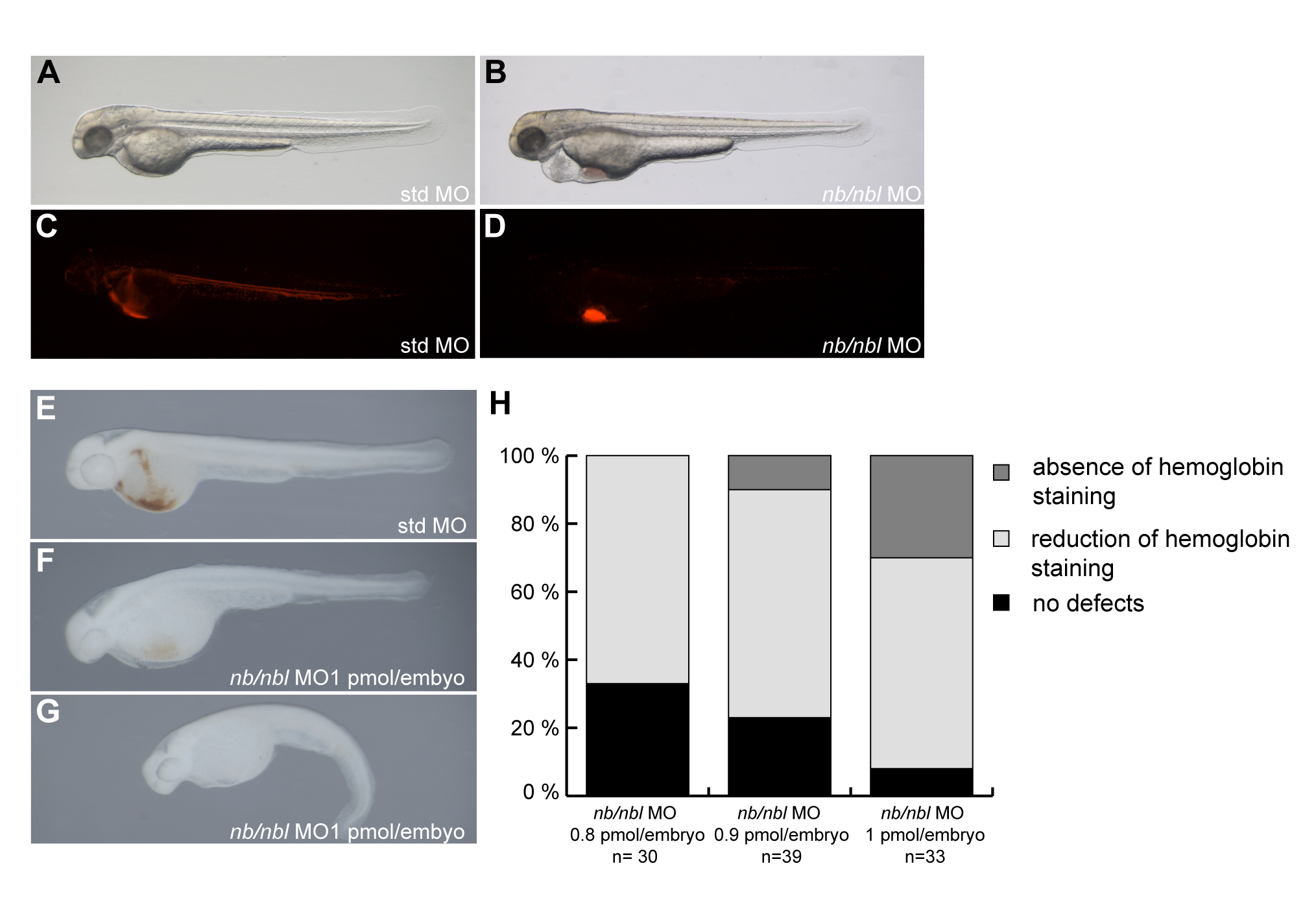

Supplement: Figure S1 — Dose dependent hematopoietic phenotype induced by nb/nbl MO. A–D. Tg(gata1:dsRed) std MO and nb/nbl MO (0.8pmol/embryo) at 48 hpf. Images were taken in bright field (A, B) and using a rhodamine emission filter (C, D). E–G. Analysis of the hemoglobin content by whole embryo o-dianisidine staining. 48 hpf std MO embryos (E) and nb/nbl morphants (1 pmol/embryo; F, G). At this dose the 62% of nb/nbl morphants shows a drastic reduction of the hemoglobin content (F), an additional 30% shows complete loss of hemoglobin staining (G). H. Injection of different doses of nb/nbl MO (0.8–1 pmol/embryo) produces a dose-dependent hematopoietic phenotype. The data are referred to a single typical experiment. (2.29 MB TIF) [file pone.0014296.s001.tif]

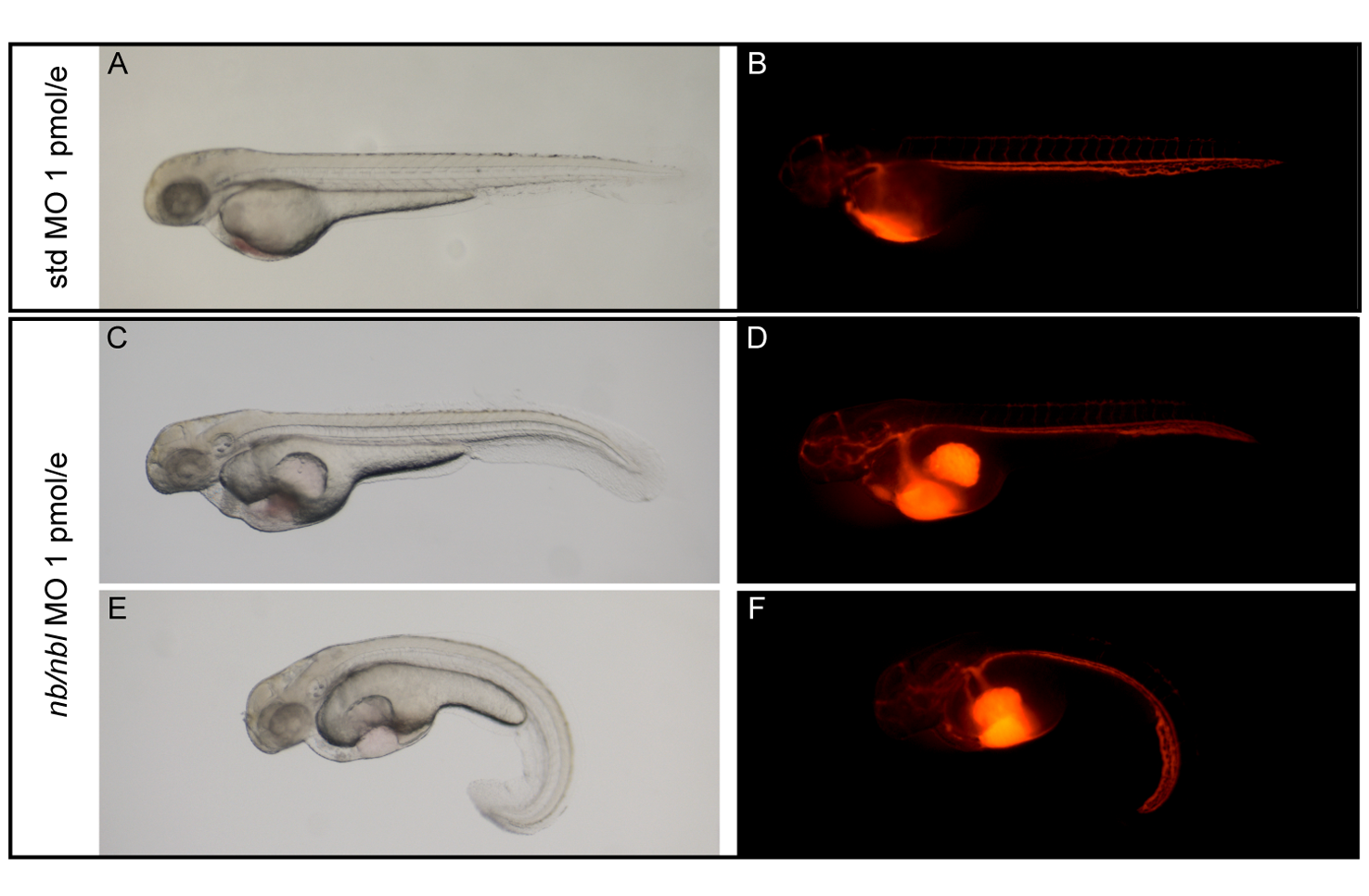

Supplement: Figure S2 — The heart functionality and the axial vasculature are not drastically compromised in nb/nbl morphants. Microangiography experiments, were performed on 2 dpf controls (A, B) and embryos injected with nb/nbl MO at the high dose of 1 pmol/embryo (C–F). In nb/nbl morphants (D, F) the injected dye flows into the main axial vessels as in control embryos (B). (1.06 MB TIF) [file pone.0014296.s002.tif]

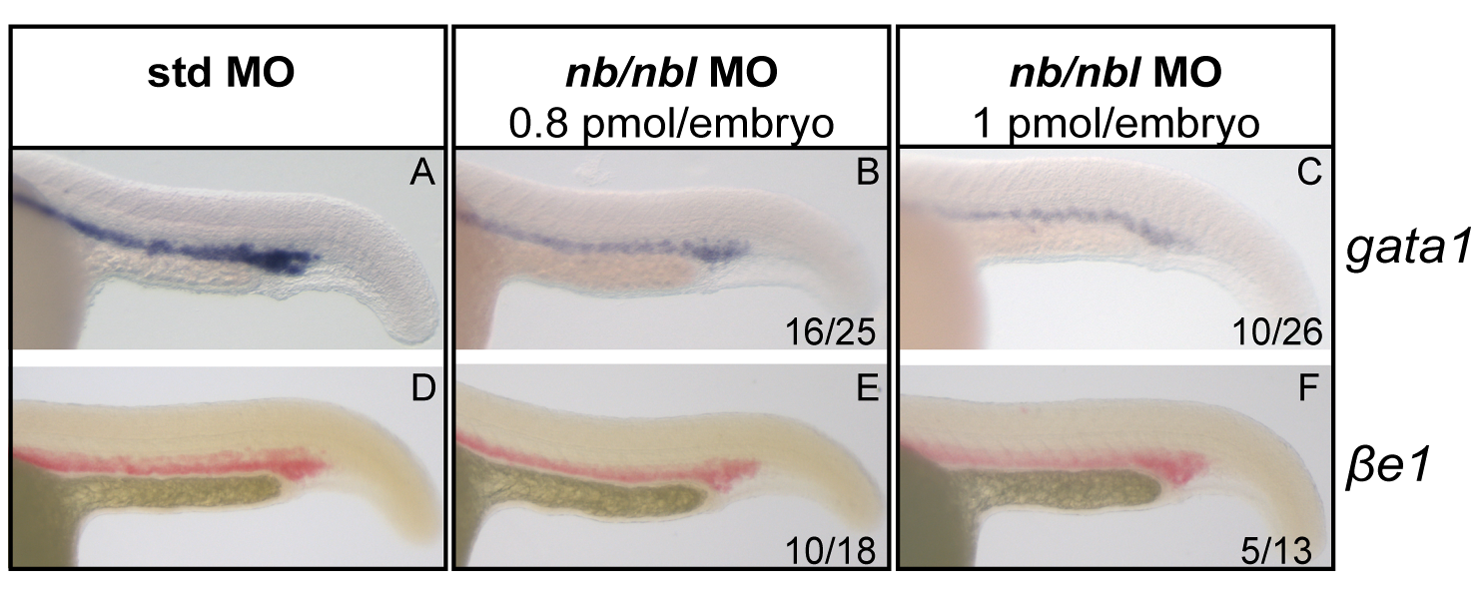

Supplement: Figure S3 — Dose dependent reduction of gata1 and βe1 globin expression in nb/nbl morphants. WISH were performed on controls (std MO; A, D) and embryos injected with different doses of nb/nbl MO (0.8 pmol/embryo, B, E; 1 pmol/embryos, C, F). In the ICM of 22–24 hpf nb/nbl MO-injected embryos the downregulation of gata1 and βe1 appears dose dependent. (0.86 MB TIF) [file pone.0014296.s003.tif]

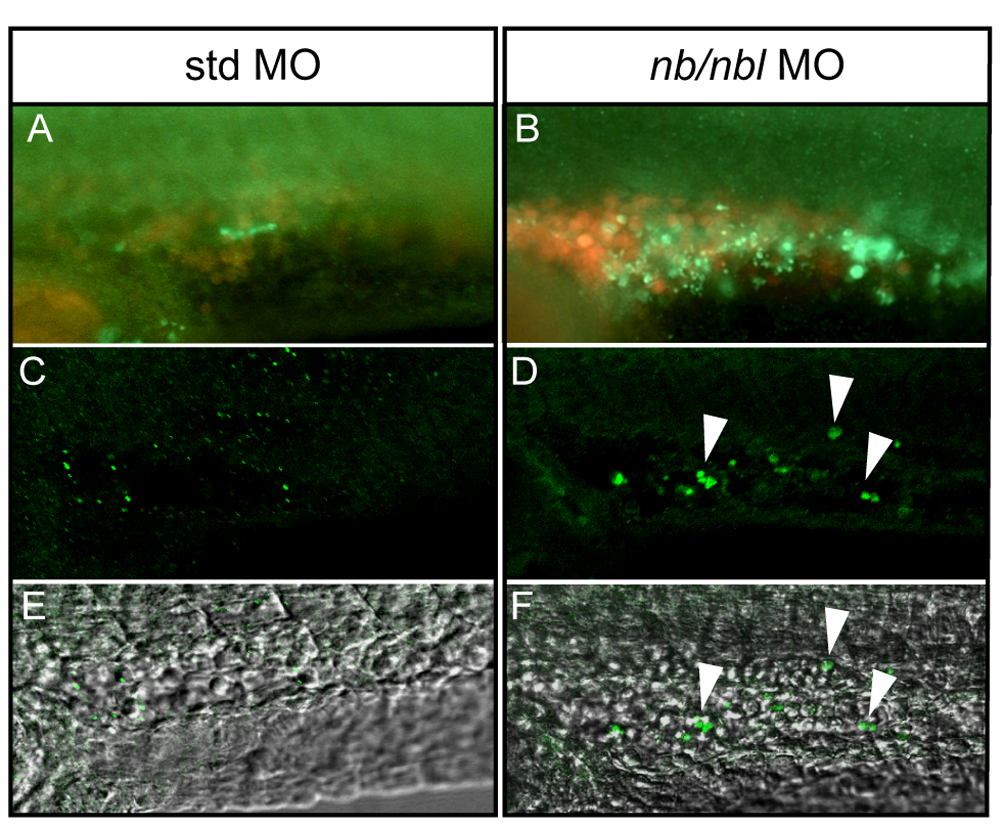

Supplement: Figure S4 — Caspase-3 activation in nb/nbl morphants at 28–30 hpf. A–B. Whole-mount immunofluorescence to detect caspase-3 activation (green signal), detailed view of the ICM region of 26–28 hpf Tg(gata1:dsRed) embryos injected with std MO (A) and nb/nbl MO (B). C–F. Single optical sections of 26–28 hpf control and nb/nbl MO-injected embryos in which whole-mount immunofluorescence for caspase-3 activation (green signal) was performed. Fluorescent images (C, D) were merged with bright field images (E, F). Detailed view of the ICM region. Caspase-3 activation can be detected in erythroid cells of nb/nbl morphants (white arrowheads; D, F). (1.15 MB TIF) [file pone.0014296.s004.tif]

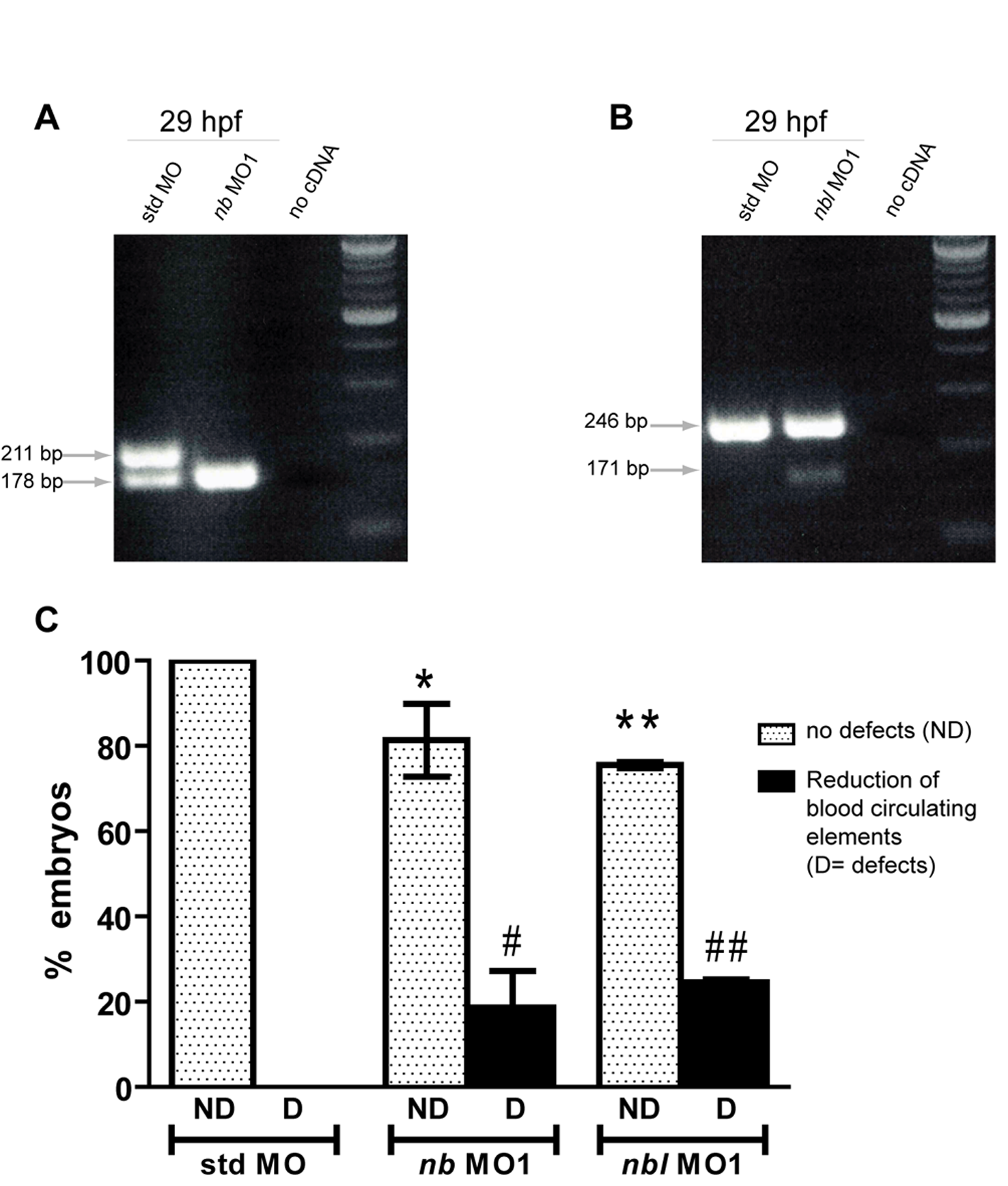

Supplement: Figure S5 — Single numb and numblike knockdown reproduce the nb/nbl morphants hematopoietic defects with low penetrance. A–B. Injection of nb MO1 and nbl MO1 specifically blocks splicing of the targeted pre-mRNAs. PCR reactions were performed on cDNAs retrotranscribed from total RNA extracted from 29 hpf nb MO1 injected embryos (1.4 pmol/embryo; A), nbl MO1 injected embryos (0.3 pmol/embryo; B), std MO injected embryos (0.3 pmol/embryo or 1.4 pmol/embryo; A, B). β-actin has been tested as an internal control (data not shown). A control PCR reaction performed without cDNA is shown in lane 3 of both the boxes (A, B). Primers: nb MO1-5′: CACCAGTGGCAGACCGATGAA nb MO1-3′: ACCGCTCGCACAGCCTTCTTA nbl MO1-5′: TCGGGCTGGTGGAGGTGGAT nbl MO1-3′: CCGTCACGGCAGATGTAAGAG. C. Single injection of nb MO1 (1.4 pmol/embryo) or nbl MO1 (0.3 pmol/embryo) in Tg(gata1:dsRed) produces the hematopoietic phenotype respectively in ∼19% (n = 99) and ∼25% (n = 125) of the MO injected embryos (*p<0.05 vs std MO no defects, **p<0.01 vs std MO no defects, #p<0.05 vs std MO defects, ##p<0.01 vs std MO defects). 100% of control embryos was unaffected (n = 65). (0.95 MB TIF) [file pone.0014296.s005.tif]

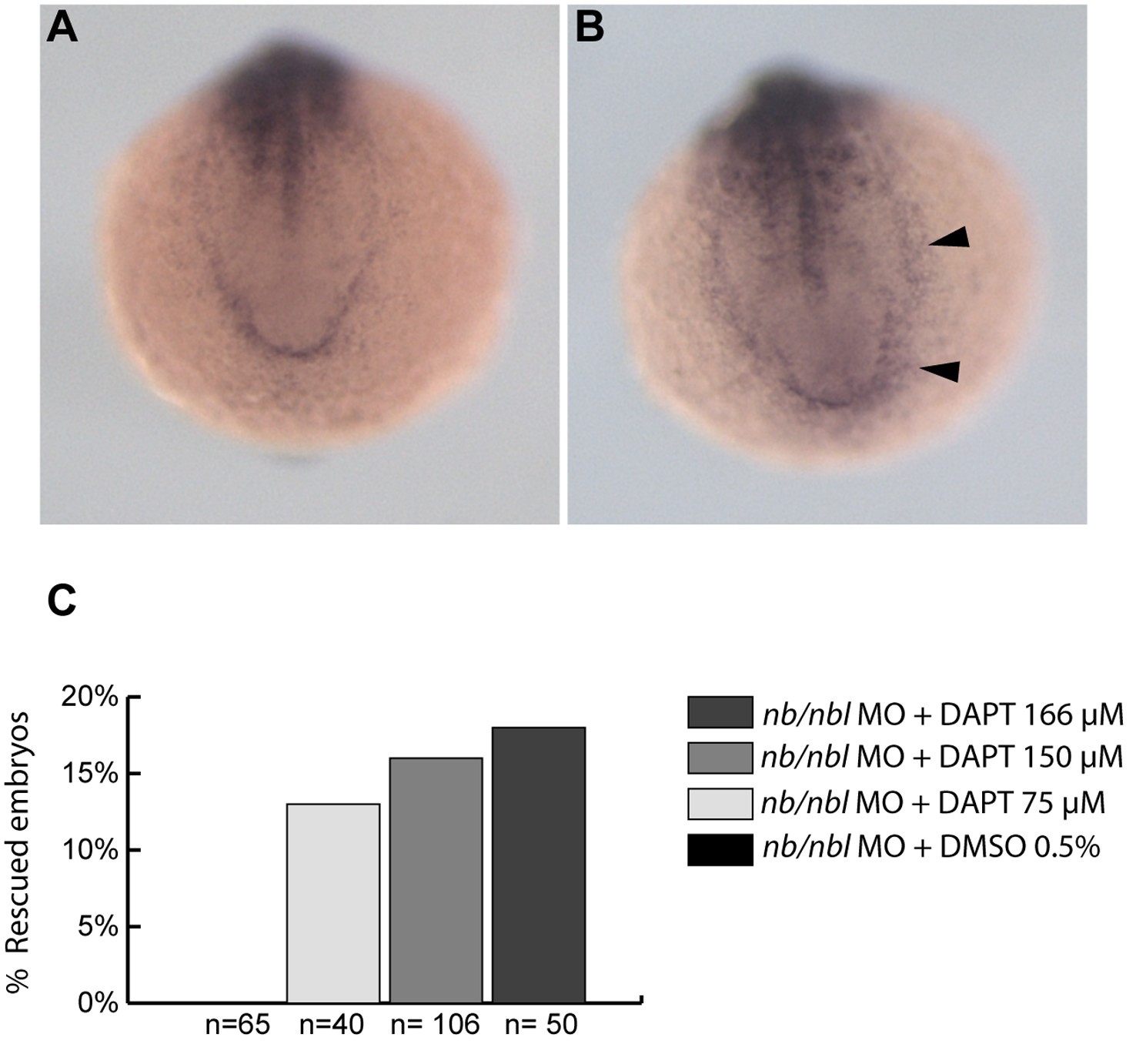

Supplement: Figure S6 — her6 is ectopically expressed in nb/nbl morphants. A–B. Posterior view of 8–10-ss embryos. The nb/nbl morphants display an enlarged expression domain of the Notch target gene her6 within the PLM region (B; black arrowheads), when compared to controls (A). C. Percentages of rescue of the hematopoietic phenotype in nb/nbl morphants treated with different concentrations of DAPT. (1.00 MB TIF) [file pone.0014296.s006.tif]
